# Effect of traditional chinese medicine (TCM) and its fermentation using *Lactobacillus plantarum* on ceftriaxone sodium-induced dysbacteriotic diarrhea in mice

**DOI:** 10.1186/s13020-022-00575-x

**Published:** 2022-02-09

**Authors:** Xin Guo, Zipeng Yan, Jixiang Wang, Xinfeng Fan, Jie Kang, Ruiyan Niu, Zilong Sun

**Affiliations:** 1 College of Veterinary Medicine, Shanxi Agricultural University, Taigu, 030801 China; 2 College of Life Sciences, Shanxi Agricultural University, Taigu, 030801 China; 3 Fenyang College of Shanxi Medical University, Fenyang, 032200 China

**Keywords:** Fermented TCM, Diarrhea, Antibiotic, Sijunzi decoction

## Abstract

**Background:**

Buzhongyiqi decoction (BD), Sijunzi decoction (SD), and Shenlingbaizhu decoction (SHD) have been extensively used clinically for the treatment of diseases caused by spleen-Qi deficiency and microbial fermentation has historically been utilized in traditional Chinese medicine (TCM). This study aimed to investigate the mitigative effect of TCM and fermented TCM (FTCM) with *Lactobacillus plantarum* (LP) on antibiotic-associated diarrhea, and to select an optimal formula and then identify its compounds.

**Methods:**

Dysbacteriosis in mice was induced by ceftriaxone sodium (CS). The mice were then treated with LP, BD, SD, SHD, fermented BD, fermented SD (FSD), and fermented SHD. Diarrhea indexes, the abundances of gut bacteria, intestinal morphometrics, and mRNA expressions of genes related to intestinal barrier function were assessed. Then, ultra-high-performance liquid chromatography coupled with quadrupole time-of-flight mass spectrometry (UHPLC-Q-TOF/MS) were employed to identify and relatively quantify the compounds in the selected decoctions.

**Results:**

CS significantly increased the fecal output weight, the total number of fecal output, and fecal water content, indicating the occurrence of diarrhea. Bacterial culture tests showed that the above symptoms were accompanied by the disruption of specific intestinal flora. TCM, LP, and FTCM alleviated the diarrhea index and recovered the intestinal microbiota. FTCM showed more advantageous than TCM or LP alone. The mRNA expressions of aquaporins (AQPs) and tight junctions (TJs) decreased by CS were enhanced by TCM, LP, and FTCM. In addition, through UHPLC-Q-TOF/MS, (S)-(-)-2-hydroxyisocaproic acid, L-methionine, 4-guanidinobutyric acid (4GBA), and phenyllactate (PLA) in SD and FSD were identified and relatively quantified.

**Conclusions:**

TCM, LP, and TCM fermented with LP alleviated CS-induced diarrhea symptoms, and improved the intestinal flora and barrier function. Four compounds including (S)-(-)-2-hydroxyisocaproic acid, L-methionine, 4GBA, and PLA in FSD, which were identified by UHPLC-Q-TOF/MS, might function in modulating intestinal flora and improving villi structure.

**Supplementary Information:**

The online version contains supplementary material available at 10.1186/s13020-022-00575-x.

## Background

Antibiotics have been used to prevent and treat of various bacterial infections. In recent years, however, numerous studies have revealed that the overuse of antibiotics results in undesirable consequences, such as antibiotic resistance, pathogen overgrowth, alteration in gut microbial composition, increase in bacterial susceptibility, and the risk of repeated infections [1]. In addition, nephrotoxicity, neurotoxicity [2], emesis, antibiotic-associated diarrhea (AAD), and allergy [3] are the most common side effects of antibiotics. Notably, antibiotics can alter the compositions of normal gut microbiota and disable the gastrointestinal function in human beings and animals, also known as dysbacteriosis [4]. Because the gut microbiota plays an important role in immunity, metabolism, and endocrinology, the negative impacts of antibiotics on the microbiota can lead to diverse further health complications as obesity, allergies, and autoimmunity [4, 5].

Probiotics have been demonstrated to regulate gut microbiota and produce flora metabolites that is of benefit to health via the following mechanisms: direct antimicrobial effects, enhancement of mucosal barrier integrity, and immune modulation [6]. In addition, traditional Chinese medicine (TCM), as one of the oldest medical practices in human history which has been widely applied to clinical diagnoses and treatments [7], can also adjust the balance of intestinal flora [8]. In TCM practice, tonic herbs and animal sourced medicines can strengthen the body and cure diseases caused by Qi deficiency. There are some typical herbs commonly used for tonifying Qi, including Astragali Radix, Ginseng Radix et Rhizoma (ginseng), yam, Codonopsis, Coix seed, Atractylodis Macrocephalae Rhizoma, Glycyrrhizae Radix et Rhizoma (licorice), and Schisandra Chinensis Fructus. Besides single herbs, there also have exemplary Qi-invigorating multi-ingredient decoctions (called Tang in Chinese) such as “Sijunzi Tang, Lizhong Tang, Buzhongyiqi Tang, and Shenlingbaizhu Tang” [7]. TCMs are mostly relevant to intestinal flora because they inevitably interact with the gut microbiota through oral administration [9, 10]. Investigations further demonstrated that TCM metabolites are of importance in pharmacological activities [8]. Furthermore, microorganisms have been employed in fermenting TCM. It is well known that probiotics are used to ferment Scutellaria Radix [11], Atractylodis Macrocephalae Rhizoma [12], red ginseng [13], and some Chinese prescriptions such as Danggui Buxue Tang [14], Sagunja-tang [15], Ge-Gen-Qin-Lian decoction [16].

In TCM, the single herbs were usually combined into the form of herbal formulas which were used to therapy various clinical diseases through a multi-component, multi-target and multi-pathway approach [17, 18]. Therefore, in this study, three classic prescriptions, Buzhongyiqi decoction (BD), and Sijunzi decoction (SD), and Shenlingbaizhu decoction (SHD), were selected to investigate their anti-diarrhea effects on AAD in mice. In addition, SD and fermented SD, owing to their better effects, were got further tests to identify and relatively quantify the compounds through untargeted ultra-high-performance liquid chromatography coupled with quadrupole time-of-flight mass spectrometry (UHPLC-Q-TOF/MS).

## Materials and methods

### Medicines and strain

All herbal constituents of BD, SD, and SHD were purchased from Beijing Tong Ren Tang Co., Ltd. (Beijing, China). Ceftriaxone sodium (CS) was purchased from Beijing Solarbio Science & Technology Co., Ltd. (Beijing, China), and *Lactobacillus plantarum* (LP) CICC 21,809 was obtained from the China Center of Industrial Culture Collection (CICC, Beijing, China).

Three decoctions were prepared according to the *Pharmacopoeia of the People’s Republic of China*. In briefly, the original composition of SD, namely, Radix Codonopsis (60 g), Atractylodis Macrocephalae Rhizoma (60 g), Poria (60 g) and Glycyrrhizae Radix et Rhizoma Praeparata (30 g), were soaked in distilled water at room temperature for 1 h, and then decocted twice in 2,100 mL of distilled water for 1 h per time [19], and finally concentrated to 1 g/mL by a rotary evaporation apparatus (RE3000-A, Shanghai Yarong Biochemical Instrument Factory, China). BD is composed of the following eight herbs: Radix Astragali (75 g), Codonopsis Radix (60 g), Atractylodes Macrocephala Koidz (60 g), Glycyrrhizae Radix et Rhizoma Praeparata (30 g), Angelicae Sinensis Radix (30 g), Citrus Reticulata (20 g), Cimicifugae Rhizoma (20 g), Radix Bupleuri (20 g). SHD is composed of 11 herbs: Codonopsis Radix (60 g), Poria (30 g), Atractylodes Macrocephala Koidz. (60 g), Rhizoma Dioscoreae (60 g), Glycyrrhizae Radix et Rhizoma Praeparata (30 g), Lablab Semen Album (60 g), Lotus Seed (30 g), Coicis Semen (30 g), Fructus Amomi (15 g), Platycodon Grandiforus (30 g), and Citrus Reticulata (30 g). These herbs for SD and SHD were decocted in 3150 mL or 4350 mL of distilled water, respectively, and concentrated as described above. The compositions of BD, SD, and SHD are shown in Table 1.

**Table 1:** The herb compositions

Name of TCM	Place of production
Radix Astragali	Gansu
Codonopsis Radix	Shanxi
Atractylodes Macrocephala Koidz.	Zhejiang
Glycyrrhizae Radix et Rhizoma Praeparata	XinJiang
Angelicae Sinensis Radix	Gansu
Citrus Reticulata	Zhejiang
Cimicifugae Rhizoma	Neimenggu
Radix Bupleuri	Shanxi
Poria	Hubei
Lablab Semen Album	An’hui
Rhizoma Dioscoreae	Henan
Lotus Seed	Hubei
Coicis Semen	Guizhou
Platycodon Grandiforus	An’hui
Fructus Amomi	Guangdong

The LP strain was inoculated into sterile liquid MRS medium for 24 h at 37 °C, then, inoculum LP (3%, v/v) was inoculated into BD (100 mL), SD (100 mL), and SHD (100 mL), respectively, in which the pH was adjusted to 6.5 using food-grade Na_2_CO_3_. The fermented decoctions inoculated with LP were incubated at 37 °C for 24 h to reach concentration of 10^9^ colony forming units (CFUs) mL^−1^. Non-fermented TCM decoctions were kept at 4 °C and heated to 37 °C before being administered to mice via gavage.

### Animals and experimental design

72 healthy 3-week-old male ICR mice were obtained from the Experimental Animal Center of Shanxi Medical University (Taiyuan, Shanxi, China). All mice were maintained under specific pathogen-free conditions and fed with sufficient food and water, and the environment was maintained at 22 °C–25 °C, with a 12 h light/dark cycle. This animal study was approved by the Experimental Animal Ethics Committee of Shanxi Agricultural University (Taigu, Shanxi, China; Approval number: SXAU-EAW-2019M0613) and performed in accordance with their guidelines.

After 1-week of acclimatization, mice were randomly divided into nine groups (n = 8). In the blank control (BC) group, mice were treated with 0.3 mL of saline intragastrically twice per day at 09:00 and 13:00. In the CS group, mice were given 0.3 mL of CS at the dose of 4 g/kg per day for 7 days at 09:00 and saline at 13:00 by gavage, respectively. For the CS + LP group, the CS + BD group, the CS + SD group, the CS + SHD group, the CS + fermented BD (CS + FBD) group, the CS + fermented SD (CS + FSD) group, and the CS + fermented SHD (CS + FSHD) group, mice were given 0.3 mL of CS at 09:00 and 0.3 mL of corresponding decoctions at 13:00 by gavage for 7 days, respectively. On 1st day, 4th day, and 7th day, diarrhea symptoms were recorded and fresh stool samples from mice in each group were collected for live bacterial culture. On 8th day, the mice were euthanized. The intestinal tissues were collected and stored at −80 °C for quantitative real-time polymerase chain reaction (qRT-PCR). Three samples from each group were fixed in 4% paraformaldehyde solution for intestinal histopathology.

### Diarrhea assessment and live bacterial culture

All mice were kept separately, with one mouse per cage. 2 h after gavage at 13:00, diarrhea symptoms were assessed by three indicators: fecal output weight [20], total number of fecal output [21], and fecal water content [20]. Fecal output was determined by measuring cumulative stool weight. Total number of fecal output in each group was obtained by counting the fecal numbers of mice. Fecal samples were weighed and dried, and then the dried solid weight and total fecal weight were measured. The fecal water content was calculated by the equation: fecal water content = 1 − (dried solid weight)/(total fecal weight).

Fresh feces were homogenized and serially diluted in saline solution. Aliquots with the dilutions of 10^−2^ to 10^−7^ were then spread onto different selective agar plates. Lactobacillus selective (LBS), tryptone-yeast extract (TPY), bile esculin azide (BEA), and eosin-methylene blue (EMB) agar medium (Solarbio, Beijing, China) were used to detect *Lactobacillus*, *Bifidobacterium*, *Enterococcus* and *Colibacillus*, respectively. The plates were incubated at 37°C in an anaerobic or aerobic atmosphere for 48 h.

### Intestinal morphometrics

The fixed tissues of duodenum, jejunum, and ileum were cut into a tube of about 1 cm along the cross-section, then dehydrated across an ethanol gradient, cleared with xylene, embedded in paraffin wax, and sectioned at a thickness of 4 μm. The intestinal tissues were stained with hematoxylin and eosin (HE). Images were observed under an Olympus BX51 microscope equipped with CCD DP70 video camera (Olympus Optical, Tokyo, Japan). The villus height and crypt depth were measured using Image-Pro Plus (Version 5.1, Media Cybernetics, Silver Spring, MD, USA), and the ratio of the villus height to crypt depth (VH/CD) was calculated.

### RNA extraction and qRT-PCR

After 0.5–1 cm duodenum and colon tissues were cut into pieces, the pieces were added to 1 mL of TRIzol (Takara, Dalian, China) for RNA extraction. The quality of RNA was examined by NanoDrop ND-2000 Spectrophotometer (Nano-Drop, USA). Afterward, total RNA was reversely transcribed at 37 °C for 15 min, 85 °C for 5 s and 4 °C for 10 min using a PrimeScript™ kit (Takara, Dalian, China). The primers of *GAPDH*, *AQP1*, *AQP3*, *AQP4*, *ZO-1*, and *Occludin*, designed by Primer 3.0 plus, are shown in Table 2. qRT-PCR was performed by Agilent Mx3000P QPCR (Stratagene, USA) using the SYBR® Premix Ex Taq™ II Kit (Takara, Dalian, China). The qRT-PCR cycling conditions were as follows: 95 ℃ for 30 s, 95 ℃ for 5 s, 60 ℃ for 30 s, 72 ℃ for 30 s, 95 ℃ for 15 s, 60 ℃ for 1 min, 95 ℃ for 15 s, and repeated for 40 cycles. *GAPDH* was utilized as internal control, and the expression levels of the target gene were assessed using the 2^−ΔΔ Ct^ method.

**Table 2:** Primer sequences for qRT-PCR

Gene	Primer sequences (5′→3′)	Product (bp)	Accession no.
*GAPDH*	Forward: TGTGGATCAGCAAGCAGGAG Reverse: ACGCAGCTCAGTAACAGTCC	87	NM_001289726.1
*AQP1*	Forward: TTCCTGGTCTCAGAGCTTCC Reverse: TGGGTCCCTCACTTTCACTC	91	NM_007472.2
*AQP3*	Forward: CTTGTGATGTTTGGCTGTGG Reverse: AAGCCAAGTTGATGGTGAGG	85	NM_016689.2
*AQP4*	Forward: GTGTCTGTGGCAGCGAGATA Reverse: GCATCTGCCTCAGAACATGA	116	NM_001308641.1
*ZO-1*	Forward: TTCAGCAGCAACAGAACCAG Reverse: CGATCGTCATGCAAATCAAG	88	XM_006540786.4
*Occludin*	Forward: GAATGGCAAGCGATCATACC Reverse: CTGCCTGAAGTCATCCACAC	106	NM_001360536.1

### Identification of compounds in SD and FSD

The freeze-dried samples (n = 6) were crushed using a mixer mill (MM400, Retsch, Laichi, Germany) for 1.5 min at 30 Hz. Approximately 100 mg of powder was extracted overnight at 4 ℃ with 0.6 mL of 70% aqueous methanol. Following centrifugation at 10,000*g* for 10 min, the extracts were collected and filtered before UPHLC-MS/MS analysis.

The sample extracts were analyzed using a 1290 UHPLC system (Agilent Technologies, La Jolla, CA, USA) combined with quadrupole time-of-flight mass spectrometer (QTOF) 6600 (AB Sciex, Redwood City, CA, USA). The chromatographic separations were performed on an Agilent Eclipse Plus C18 column (1.8 μm, 2.1 mm × 100 mm, Milford, MA, USA) with the column temperature at 40℃. The mobile phases were pure water with 0.04% acetic acid (A) and acetonitrile with 0.04% acetic acid (B). The gradient program was performed as follows: the starting conditions of 95% A, 5% B, a linear gradient to 5% A, 95% B within 10 min, a composition of 5% A, 95% B kept for 1 min, and subsequently, a composition of 95% A, 5% B was adjusted within 0.10 min and kept for 2.9 min. The injection volume was 4 µL.

As described by Yang et al. [22], QTOF was operated using an electron spray ionization (ESI) in positive and negative ion mode and controlled by Analyst 1.6.3 software (AB Sciex, Waltham, MA, USA) to evaluate the full scan survey MS data. The ESI source operation parameters were as follows: source temperature 550 ℃; ion spray voltage (IS) 5500 V (positive ion mode)/-4500 V (negative ion mode); ion source gas I, gas II, and curtain gas were set at 50, 60, and 30.0 psi, respectively.

### Statistical analysis

All data were presented as means ± SEM. The significance between different groups was determined by analysis of variance (ANOVA) as implemented in Graph Pad Prism 6.0 (GraphPad Software Inc., San Diego, CA, USA), followed by Tukey’s test, with *p* < 0.05 being considered statistically significant.

## Result

### Disruption of intestinal flora by antibiotics-triggered diarrhea in mice and alleviation of treatments

**Fig. 1:**
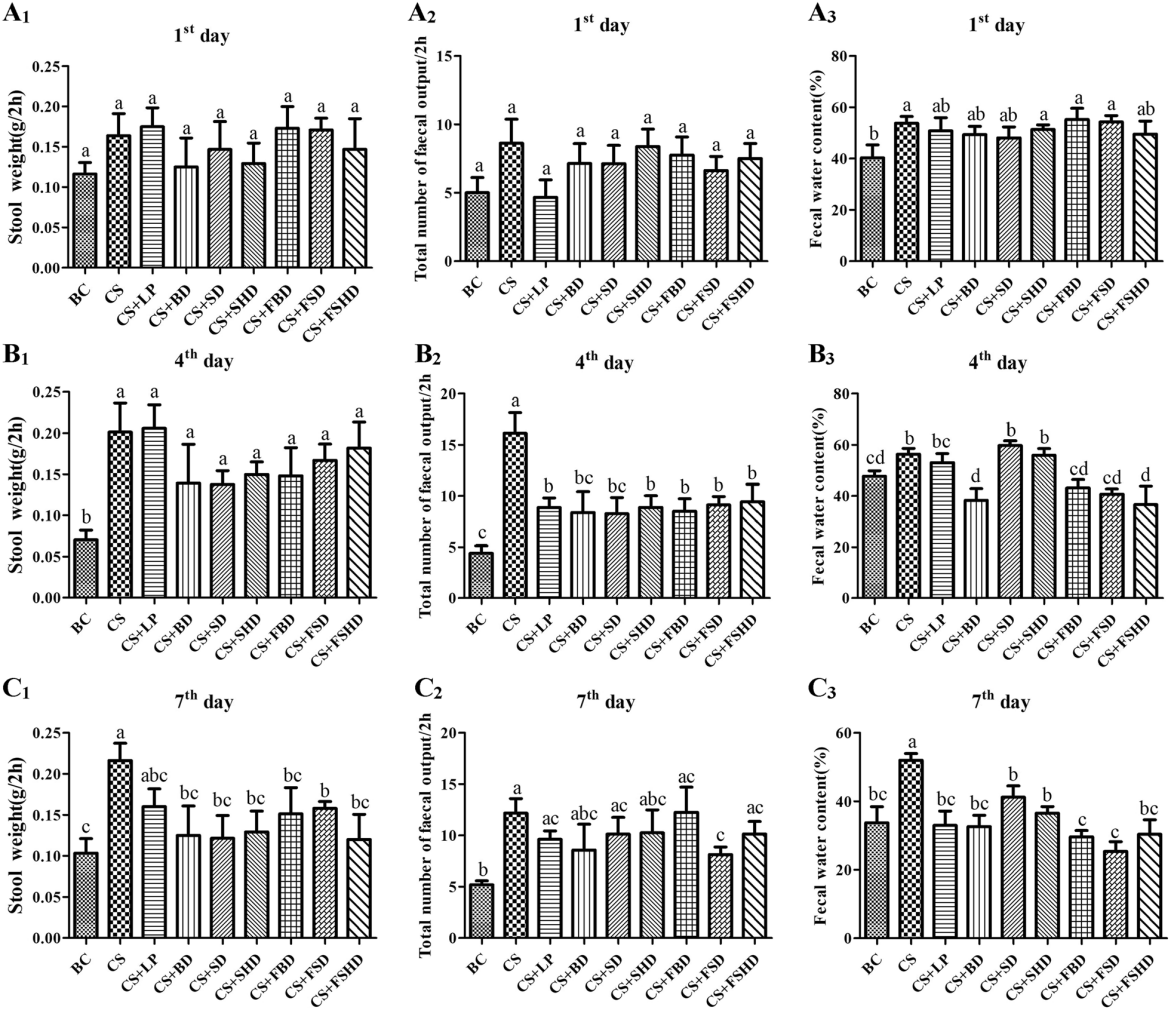
Stool weigh, total number of fecal output and fecal water content in 2 h of 1st day (**A**), 4th day (**B**) and 7th day (**C**). BC: Blank Control, CS: Ceftriaxone Sodium, CS + LP: Ceftriaxone Sodium + Lactobacillus Plantarum, CS + BD: Ceftriaxone Sodium + Buzhongyiqi decoction, CS + SD: Ceftriaxone Sodium + Sijunzi decoction, CS + SHD: Ceftriaxone Sodium + Shenlingbaizhu decoction, CS + FBD: Ceftriaxone Sodium + fermented Buzhongyiqi decoction, CS + FSD: Ceftriaxone Sodium + fermented Sijunzi decoction and CS + FSHD: Ceftriaxone Sodium + fermented Shenlingbaizhu decoction. n = 8. ^a, b, c, d^Labeled means in the bars without a common letter were significantly different (*p* < 0.05)

To investigate the effects of TCM and fermented TCM (FTCM) on AAD, the related indexes were detected in BC, CS, CS + LP, CS + BD, CS + SD, CS + SHD, CS + FBD, CS + FSD, and CS + FSHD groups. Figure 1 presents the data on stool weight, total number of fecal output and fecal water content. Compared to the BC group, the CS group showed a significant increase in fecal water content on 1st day (*p* < 0.05) (Fig. 1A_1_). Notably, on 4th day and 7th day, the CS group exhibited a significant increase in stool weight, total number of fecal output, and fecal water content compared to the BC group (*p* < 0.05) (Fig. 1B and C), indicating the occurrence of diarrhea. Meanwhile, compared to the CS group, the total number of fecal output was significantly decreased in the seven treatment groups (*p* < 0.05), and the fecal water content significantly decreased in the CS + BD, CS + FBD, CS + FSD, and CS + FSHD groups (Fig. 1B_2,3_). As shown in Fig. 1C, the stool weight and fecal water content significantly decreased in the seven treatment groups (*p* < 0.05), indicating an alleviation of symptoms by TCM, LP, and FTCM. In the CS + FSD group, the stool weight, total number of fecal output, and fecal water content were remarkably decreased (*p* < 0.05) from 4th day to day 7th day compared to the CS group. However, this was not observed in the CS + SD and CS + LP groups (Fig. 1B and C), which suggests that FSD imparts a continuous therapeutic effect.

**Fig. 2:**
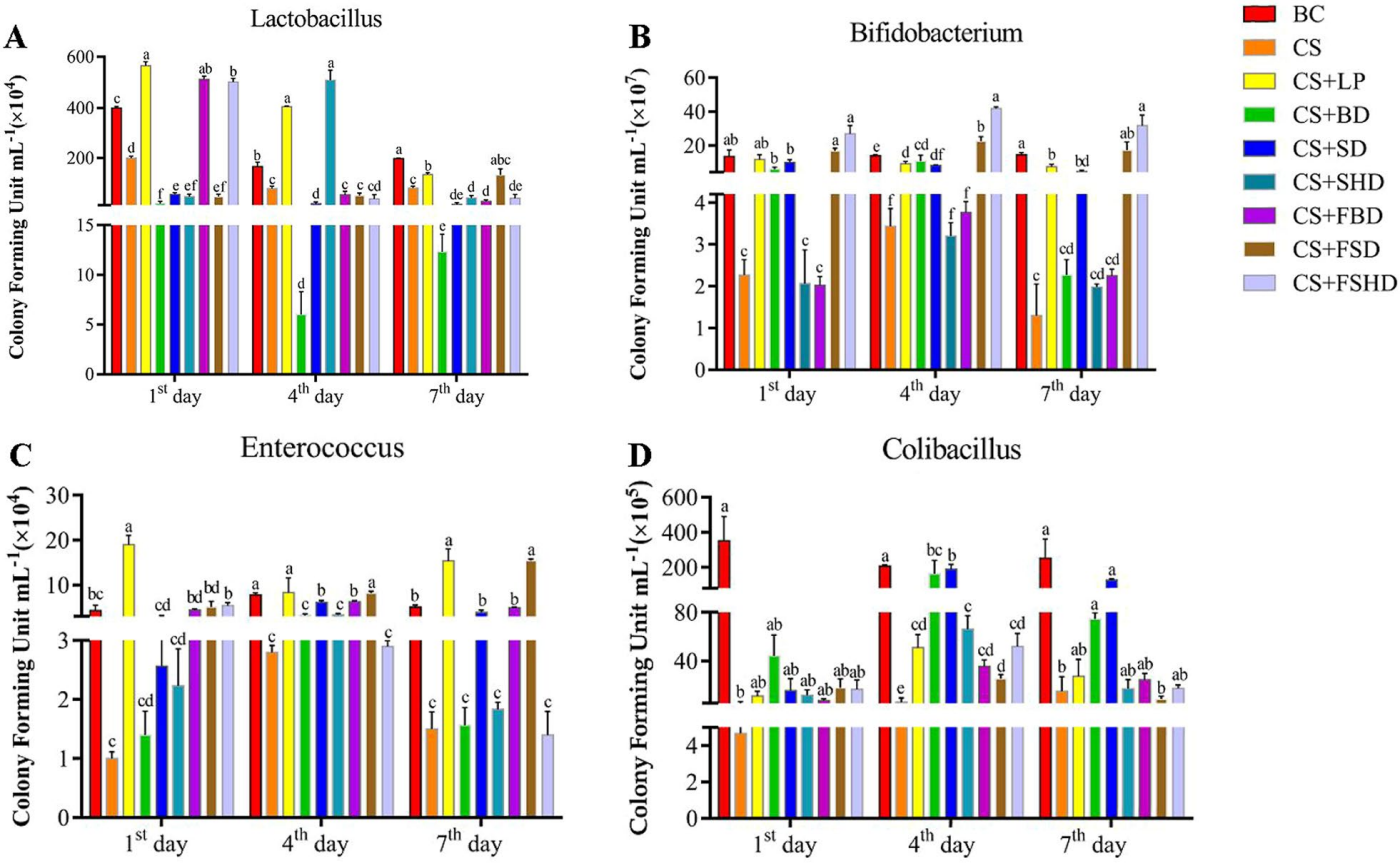
Live bacteria counting of lactobacillus (**A**), bifidobacterium (**B**), enterococcus (**C**) and colibacillus (**D**) on 1st day, 4th day and 7th day of treatments. BC: Blank Control, CS: Ceftriaxone Sodium, CS + LP: Ceftriaxone Sodium + Lactobacillus Plantarum, CS + BD: Ceftriaxone Sodium + Buzhongyiqi decoction, CS + SD: Ceftriaxone Sodium + Sijunzi decoction, CS + SHD: Ceftriaxone Sodium + Shenlingbaizhu decoction, CS + FBD: Ceftriaxone Sodium + fermented Buzhongyiqi decoction, CS + FSD: Ceftriaxone Sodium + fermented Sijunzi decoction and CS + FSHD: Ceftriaxone Sodium + fermented Shenlingbaizhu decoction. n = 3. ^a, b, c, d, e, f^Labeled means in the bars without a common letter were significantly different (*p* < 0.05)

Then, a bacterial culture test was performed to examine whether the above symptoms were accompanied by a disruption in gut bacterial composition. The results of live bacterial counting are presented in Fig. 2. Compared to the BC group, the number of lactobacilli in the feces of the CS group significantly decreased from 1st day to 7th day (*p* < 0.05). Besides the CS + LP group, there was no significant increase compared to the CS group in the other six treatment groups on 7th day (Fig. 2A). On 1st day, 4th day, and 7th day, the number of bifidobacteria in the CS group was significantly lower than that in the BC group (*p* < 0.05). Compared to the CS group, it significantly increased in the CS + LP, CS + SD, CS + FSD, and CS + FSHD groups on 7th day (*p* < 0.05) (Fig. 2B). In addition, the number of enterococci in the CS group was significantly lower than the BC group on 4th day and 7th day (*p* < 0.05). Different from bifidobacteria, the number of enterococci significantly increased in the CS + LP, CS + SD, CS + FBD, and CS + FSD groups compared to the CS group on 4th day and 7th day (*p* < 0.05) (Fig. 2C). In the CS group, a marked decrease in the abundance of colibacilli was observed on 1st, 4th and 7th days (*p* < 0.05), and other than in the CS + BD and CS + SD groups, the colibacilli abundance has not recovered on 7th day in the other treatment groups.

### Treatments improve gut barrier function in mice with CS-induced dysbacteriosis

**Fig. 3:**
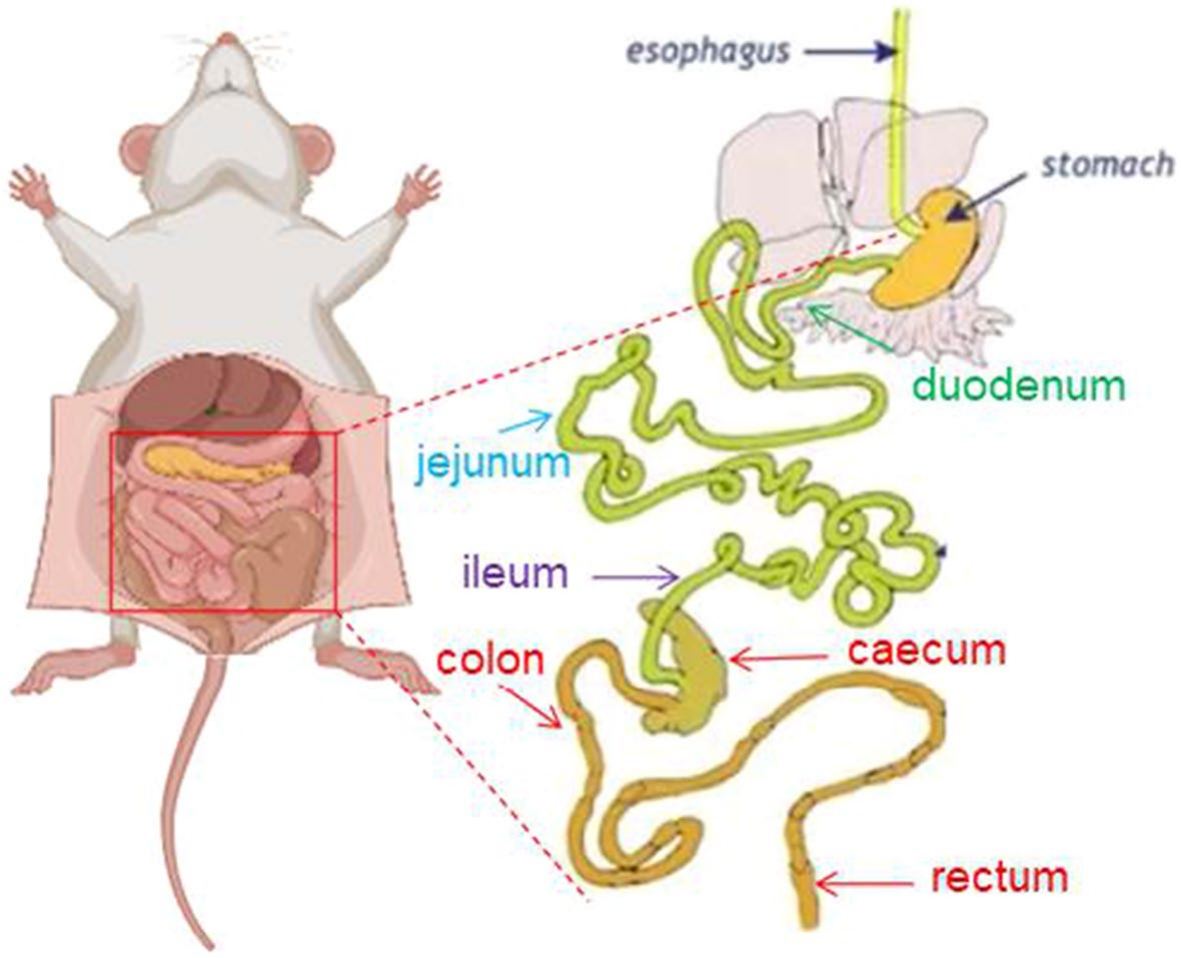
The picture of gross anatomical features of the intestines

**Fig. 4:**
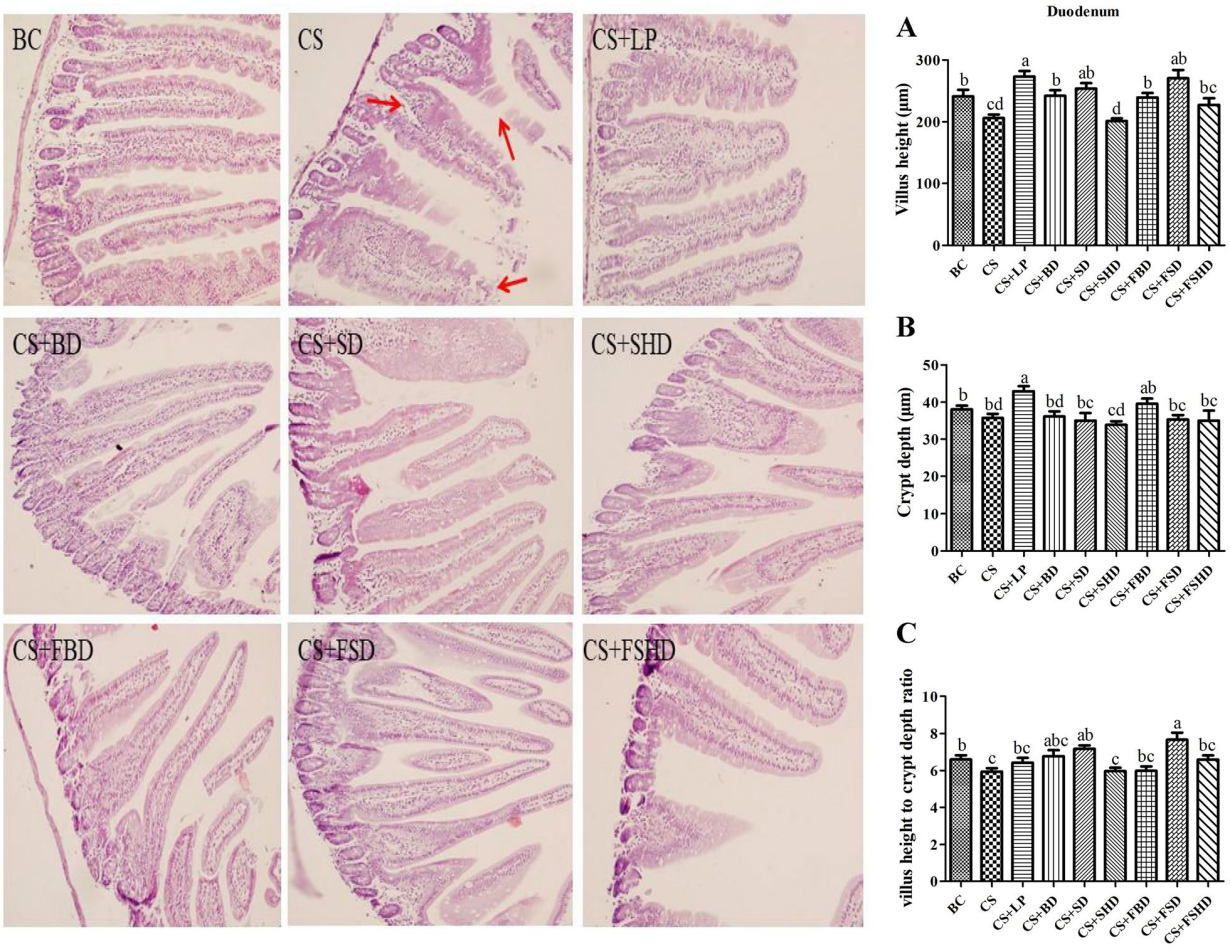
Intestinal morphology (200×) of duodenum in mice with dysbacteriosis induced by ceftriaxone sodium and the alleviation of treatments. **A** villus height, **B** crypt depth, **C** villus height to crypt depth ratio (VH/CD). BC: Blank Control, CS: Ceftriaxone Sodium, CS + LP: Ceftriaxone Sodium + Lactobacillus Plantarum, CS + BD: Ceftriaxone Sodium + Buzhongyiqi decoction, CS + SD: Ceftriaxone Sodium + Sijunzi decoction, CS + SHD: Ceftriaxone Sodium + Shenlingbaizhu decoction, CS + FBD: Ceftriaxone Sodium + fermented Buzhongyiqi decoction, CS + FSD: Ceftriaxone Sodium + fermented Sijunzi decoction and CS + FSHD: Ceftriaxone Sodium + fermented Shenlingbaizhu decoction. ^a, b, c, d^Labeled means in the bars without a common letter were significantly different (*p* < 0.05). Red arrow: villus atrophy, dissolution, damage, and epithelial cells arranged irregularly

**Fig. 5:**
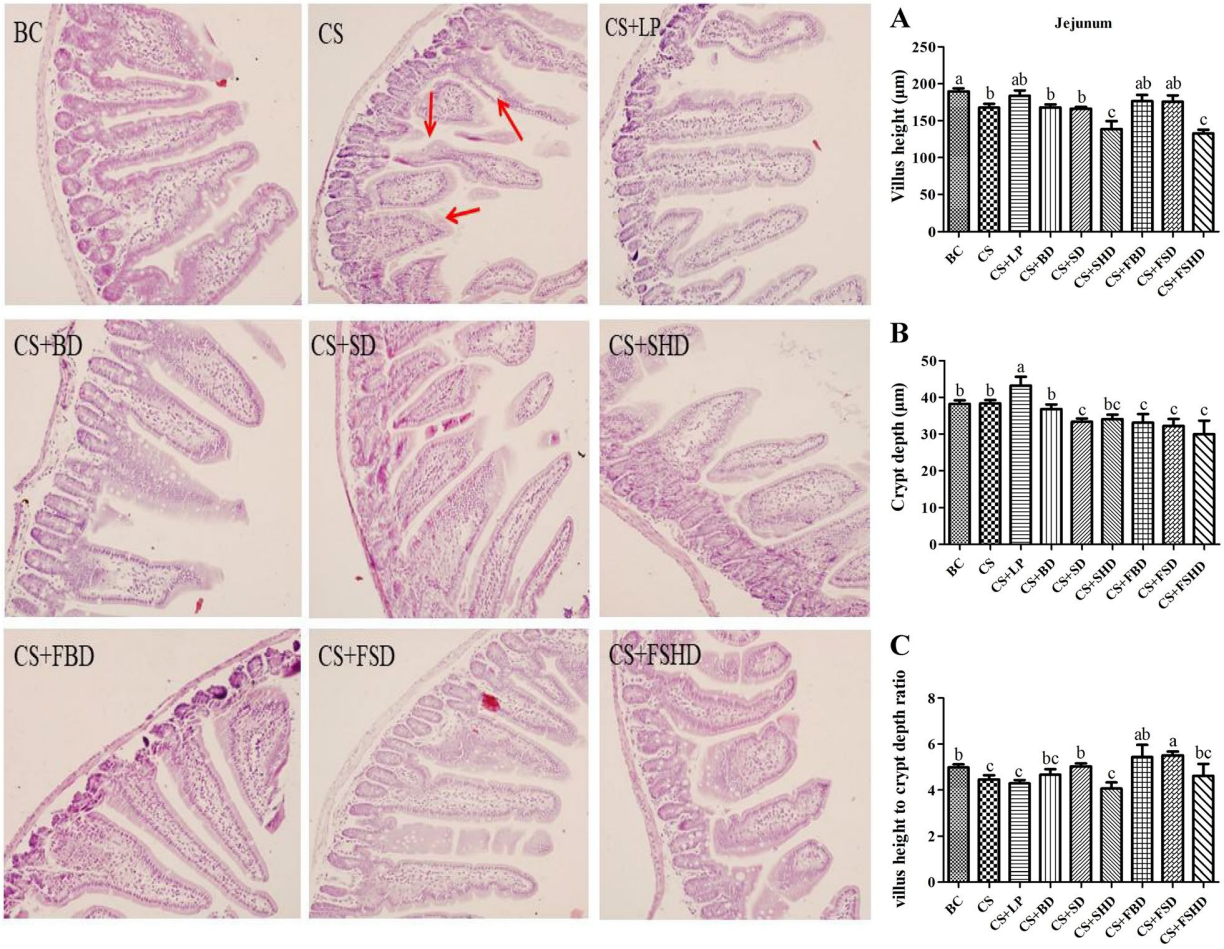
Intestinal morphology (200×) of jejunum in mice with dysbacteriosis induced by ceftriaxone sodium and the alleviation of treatments. **A** villus height, **B** crypt depth, **C** villus height to crypt depth ratio (VH/CD). BC: Blank Control, CS: Ceftriaxone Sodium, CS + LP: Ceftriaxone Sodium + Lactobacillus Plantarum, CS + BD: Ceftriaxone Sodium + Buzhongyiqi decoction, CS + SD: Ceftriaxone Sodium + Sijunzi decoction, CS + SHD: Ceftriaxone Sodium + Shenlingbaizhu decoction, CS + FBD: Ceftriaxone Sodium + fermented Buzhongyiqi decoction, CS + FSD: Ceftriaxone Sodium + fermented Sijunzi decoction and CS + FSHD: Ceftriaxone Sodium + fermented Shenlingbaizhu decoction. n = 3. ^a, b, c^Labeled means in the bars without a common letter were significantly different (*p* < 0.05). Red arrow: villus dissolution, damage, irregularly arranged epithelial cells, partially detached, and intestinal gland injury

**Fig. 6:**
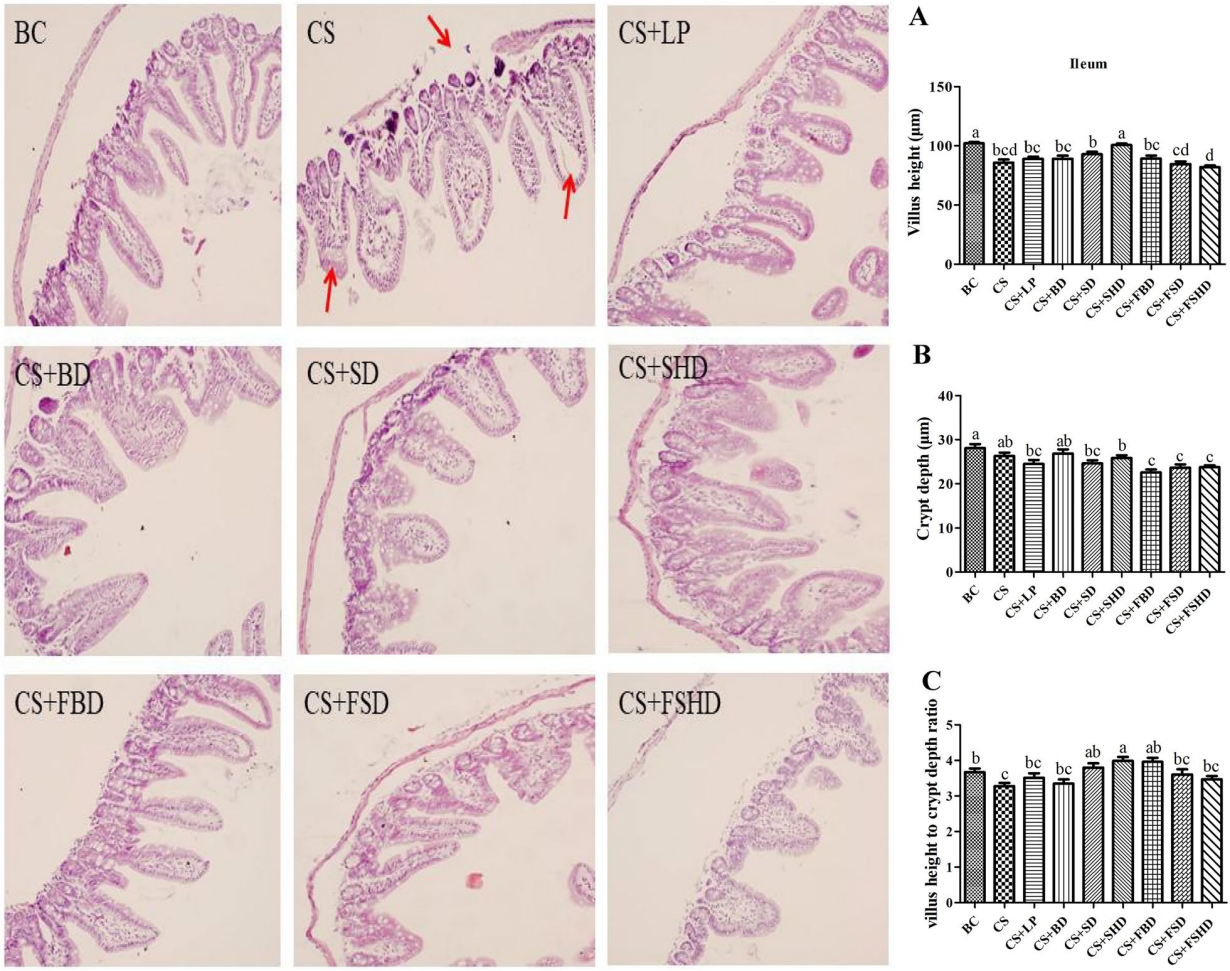
Intestinal morphology (200×) ofileum in mice with dysbacteriosis induced by ceftriaxone sodium and the alleviation of treatments. **A** villus height, **B** crypt depth, **C** villus height to crypt depth ratio (VH/CD). BC: Blank Control, CS: Ceftriaxone Sodium, CS + LP: Ceftriaxone Sodium + Lactobacillus Plantarum, CS + BD: Ceftriaxone Sodium + Buzhongyiqi decoction, CS + SD: Ceftriaxone Sodium + Sijunzi decoction, CS + SHD: Ceftriaxone Sodium + Shenlingbaizhu decoction, CS + FBD: Ceftriaxone Sodium + fermented Buzhongyiqi decoction, CS + FSD: Ceftriaxone Sodium + fermented Sijunzi decoction and CS + FSHD: Ceftriaxone Sodium + fermented Shenlingbaizhu decoction. n = 3. ^a, b, c, d^Labeled means in the bars without a common letter were significantly different (*p* < 0.05). Red arrow: the serosal layer rupture, villus dissolution, epithelial cells arranged irregularly, intestinal gland injury, and enterocyte cytoplasmic vacuolation

The picture of gross anatomical features of the intestines is shown in Fig. 3. The murine intestinal tract is comprised of a central lumen (contains the digesta) and a tube wall (includes mucous membrane, submucosa, muscle layer, and serosa). The small intestine is divided into three segments: the duodenum, jejunum, and ileum. The most proximal part of the small intestine, beginning immediately distal to the pylorus of the stomach, is the duodenum, which is associated with the pancreas and forms a U-shaped loop to the level of the umbilicus. The duodenum then transitions to the jejunum which represents the majority of the small intestine. The jejunum is slightly thicker than the duodenum. The jejunum is followed by a shorter segment of ileum which represents the terminal portion of the small intestine which connects to the cecum. The defining feature of the small intestine is the finger-like villus projections which are tallest in the duodenum and gradually decrease in length distally towards the ileum [23]. The morphology of small intestines is shown in Figs. 4, 5 and 6. The structure of duodenal, jejunal, and ileal villi in the BC group was normal, arranged neatly and evenly, and the villi and crypt were well demarcated and basically intact without obvious pathological changes. In the CS group, the duodenum, jejunum, and ileum were damaged to different degrees, as manifested by villus atrophy, dissolution, damage, irregularly arranged epithelial cells, partially detached, intestinal gland injury, and enterocyte cytoplasmic vacuolation. The intestinal tissues of mice from different treatment groups exhibited alleviation. Compared to the CS group, the degree of damage to mucosal tissues in the duodenum, jejunum, and ileum was significantly reduced in the other treatment groups. Figure 4A and C show a reduction in villi height and VH/CD in the duodenum (*p* < 0.05) in the CS group, whereas the villus height was increased in the CS + LP, CS + BD, CS + SD, CS + FBD, and CS + FSD groups (*p* < 0.05). Interestingly, the VH/CD of the duodenum was markedly increased only in the CS + SD and CS + FSD groups (*p* < 0.05). Figure 5 shows that mice with CS exhibited lower villus height and VH/CD in the jejunum than mice in the BC group (*p* < 0.05), and crypt depth was enhanced in the CS + LP group compared to the CS group (*p* < 0.05). The VH/CD significantly increased in the CS + SD, CS + FBD, CS + FSD groups compared to the CS group (*p* < 0.05). Analogously, the mice treated with CS had shorter villus height and VH/CD in the ileum than mice in the BC group (*p* < 0.05) (Fig. 6C). In contrast within the jejunum, the villus height of the ileum was enhanced in the CS + SHD group, and VH/CD was markedly improved in the CS + SD, CS + SHD, and CS + FBD groups (*p* < 0.05).

**Fig. 7:**
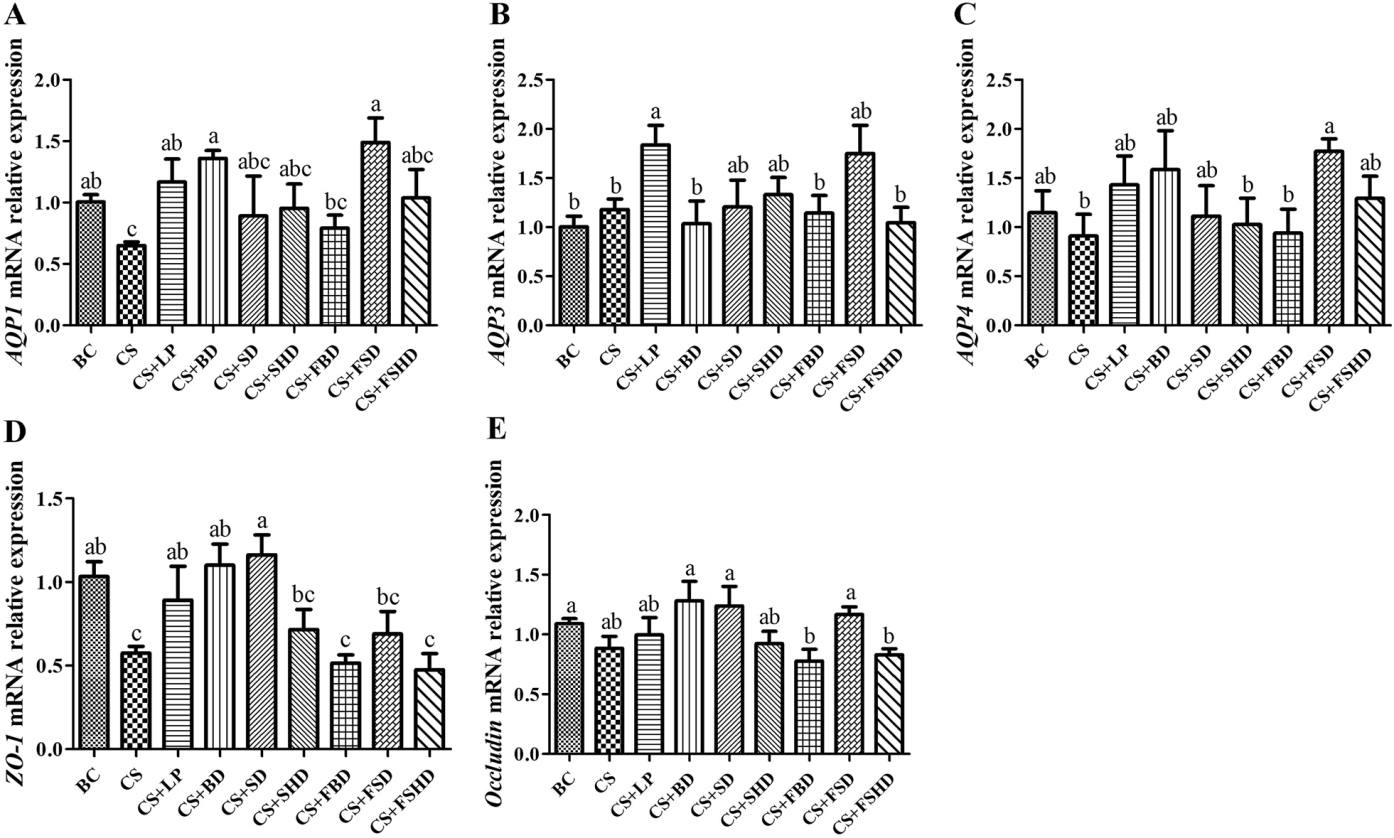
The mRNA relative expression levels of *AQP1*, *AQP3*, *AQP4*, *ZO-1*, and *Occludin* genes of duodenum in mice with dysbacteriosis induced by ceftriaxone sodium and the alleviation of treatments (**A**–**E**). AQP, Aquaporins; ZO-1, Zonula occludens-1. BC: Blank Control, CS: Ceftriaxone Sodium, CS + LP: Ceftriaxone Sodium + Lactobacillus Plantarum, CS + BD: Ceftriaxone Sodium + Buzhongyiqi decoction, CS + SD: Ceftriaxone Sodium + Sijunzi decoction, CS + SHD: Ceftriaxone Sodium + Shenlingbaizhu decoction, CS + FBD: Ceftriaxone Sodium + fermented Buzhongyiqi decoction, CS + FSD: Ceftriaxone Sodium + fermented Sijunzi decoction and CS + FSHD: Ceftriaxone Sodium + fermented Shenlingbaizhu decoction. n = 5. ^a, b, c^Labeled means in the bars without a common letter were significantly different (*p* < 0.05)

**Fig. 8:**
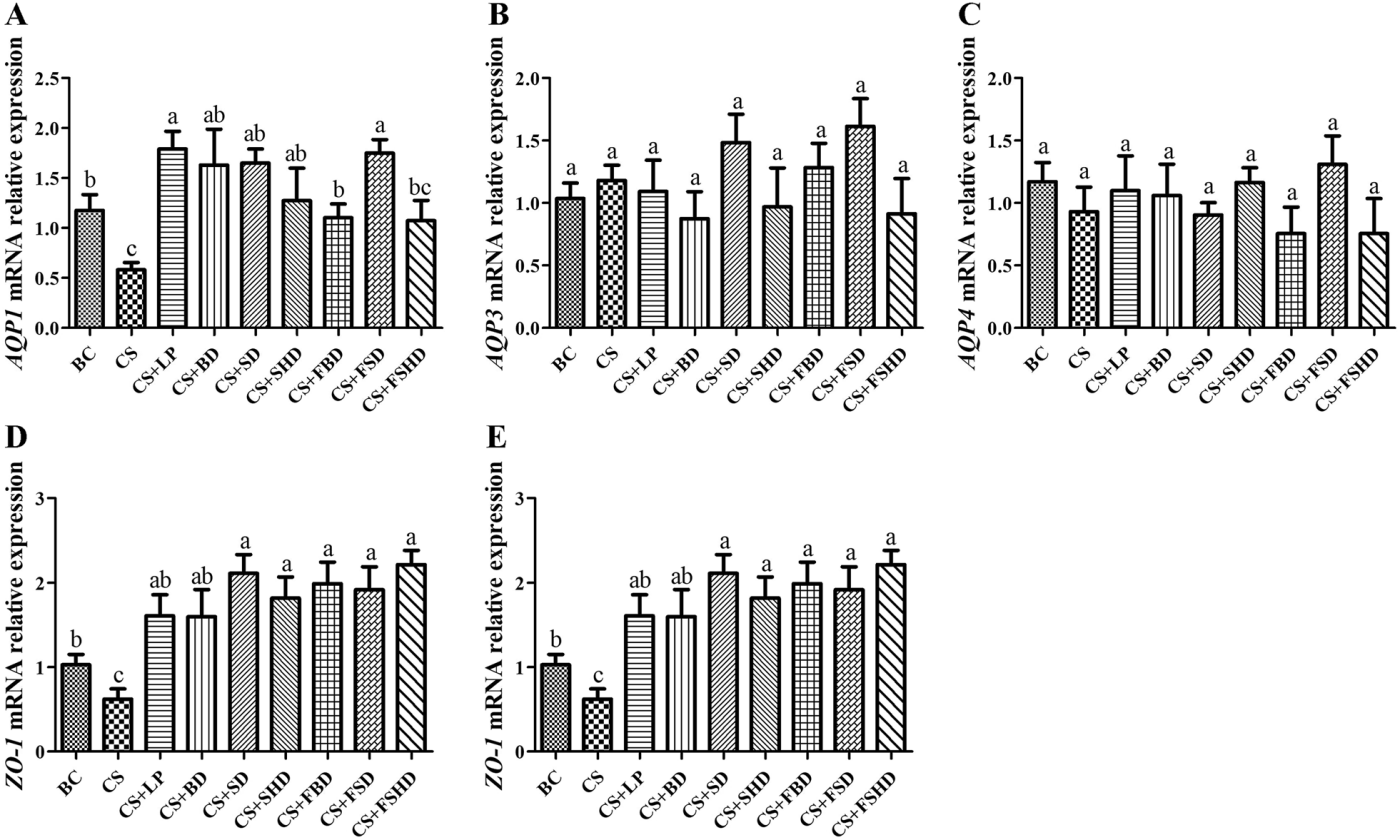
The mRNA relative expression levels of *AQP1*, *AQP3*, *AQP4*, *ZO-1*, and *occludin* genes of colon in mice with dysbacteriosis induced by ceftriaxone sodium and the alleviation of treatments (**A**–**E**). AQP, Aquaporins; ZO-1, Zonula occludens-1. BC: Blank Control, CS: Ceftriaxone Sodium, CS + LP: Ceftriaxone Sodium + Lactobacillus Plantarum, CS + BD: Ceftriaxone Sodium + Buzhongyiqi decoction, CS + SD: Ceftriaxone Sodium + Sijunzi decoction, CS + SHD: Ceftriaxone Sodium + Shenlingbaizhu decoction, CS + FBD: Ceftriaxone Sodium + fermented Buzhongyiqi decoction, CS + FSD: Ceftriaxone Sodium + fermented Sijunzi decoction and CS + FSHD: Ceftriaxone Sodium + fermented Shenlingbaizhu decoction. n = 5. ^a, b, c^Labeled means in the bars without a common letter were significantly different (*p* < 0.05)

The mRNA relative expression of aquaporins *(AQP1*, *AQP3*, and *AQP4*) and TJ proteins (*ZO-1* and *occludin*) in the duodenum and colon are presented in Figs. 7 and 8. Compared to the BC group, the mRNA expression levels of *AQP1* and *ZO-1* were markedly reduced (*p* < 0.05) in the CS gavaged mice. However, *AQP1* mRNA expression levels were enhanced in the CS + LP, CS + BD, and CS + FSD groups compared to the CS group (*p* < 0.05). In addition, *ZO-1* expression was markedly increased in the CS + LP, CS + BD, and CS +SD groups compared to the CS group (*p* < 0.05). Furthermore, compared to the BC group, the mRNA expression levels of *AQP4* did not significantly decrease in the CS group, compared to the CS group, but was enhanced in the CS + FSD group (*p* < 0.05) (Fig. 7).

As seen in Fig. 8, the mRNA expression levels of *AQP1* and *ZO-1* increased (*p* < 0.05) in the other treatment groups compared to the CS group. In addition, the *occludin* mRNA expression levels were significantly higher in the CS + BD, CS + SD, CS + SHD, and CS + FSD groups than in the CS group (*p* < 0.05). However, compared to the BC group, the changes in *AQP3* and *AQP4* mRNA expression were not significant in the other treatment groups.

### Identification of compounds in SD and FSD

**Fig. 9:**
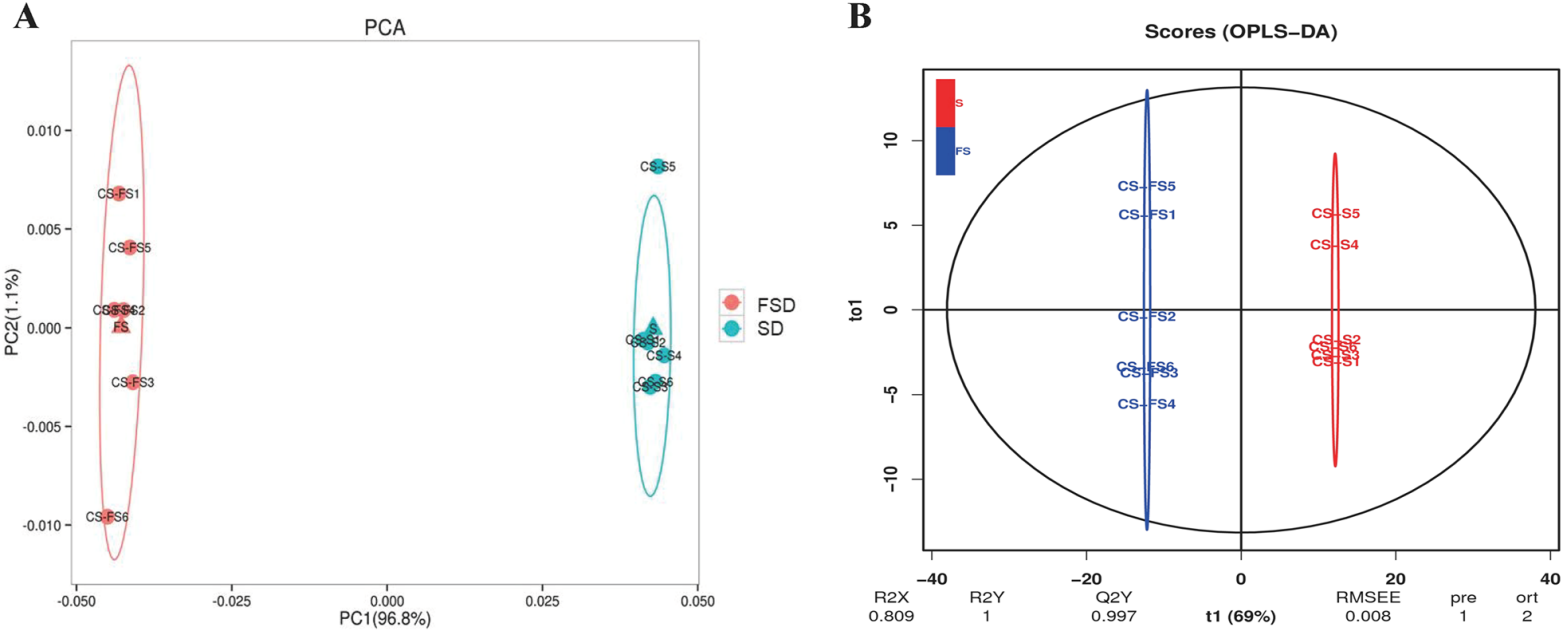
The score plots of Principal Component Analysis (PCA) (**A**) and Orthogonal Partial Least Squares-Discriminant Analysis (OPLS-DA) (**B**) of Sijunzi decoction (SD) and fermented Sijunzi decoction (FSD)

A principal component analysis (PCA) model was constructed (Fig. 9A). The aggregation trend of the two groups was separated in this model, and the spots were clustered. To reveal the significant compounds that are distinct between SD and FSD, an orthogonal partial least squares-discriminant analysis (OPLS-DA) model was established. An obvious separation trend between the two groups was observed (Fig. 9B), and the interpretation rates of the model to the X and Y matrix parameters were R^2^X = 0.809 and R^2^Y = 1, meanwhile, the prediction ability Q^2^Y = 0.997 indicated that the model had excellent reliability and prediction.

**Fig. 10:**
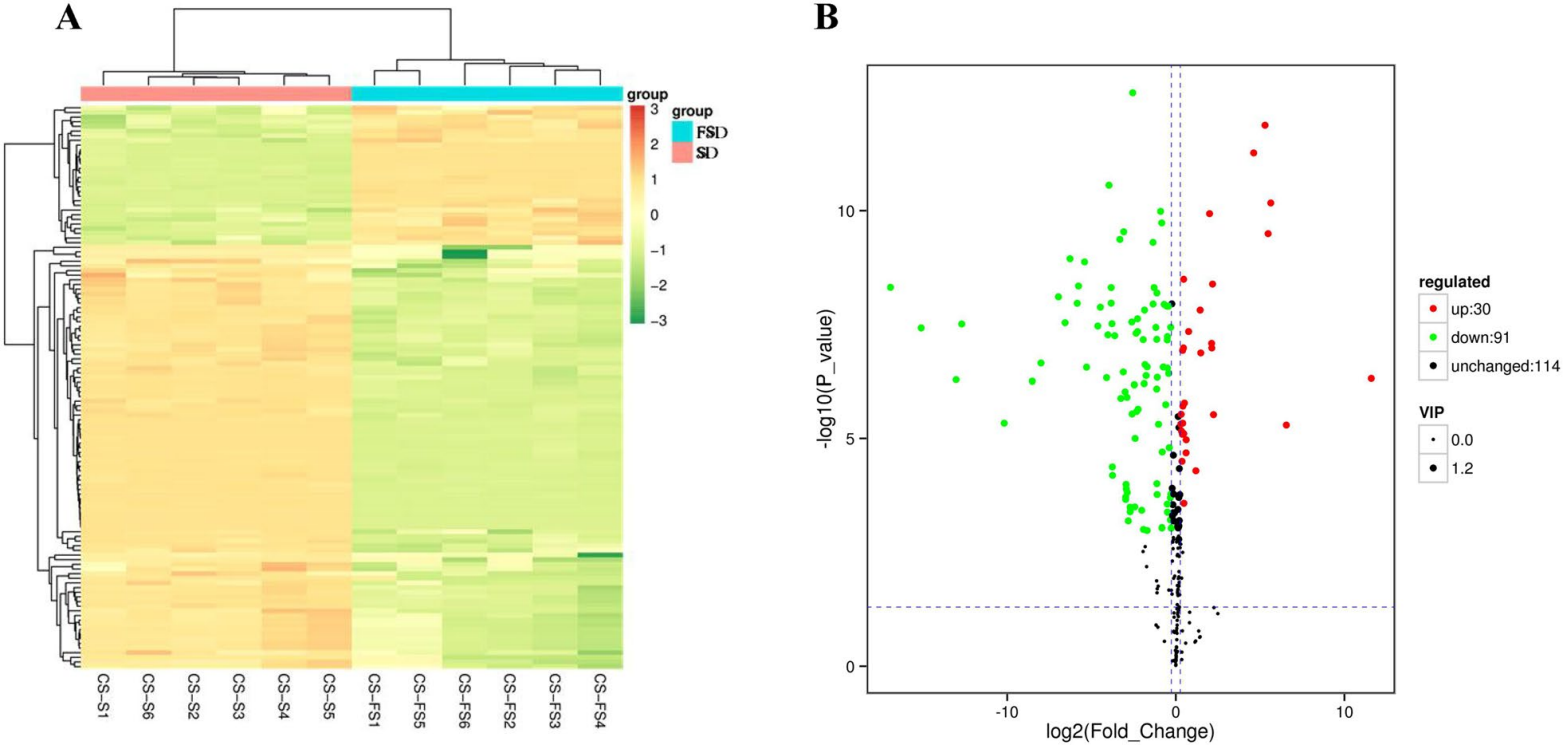
The heat maps (**A**) and volcano plots (**B**) of significantly different compounds in Sijunzi decoction (SD) and fermented Sijunzi decoction (FSD). The red spots indicate significantly up-regulated compounds, and the green spots represent significantly down-regulated compounds

Heat map analysis (Fig. 10A) showed the distribution of differential compounds in the SD and FSD groups. Differential compounds were screened out (FC > 1.2, *p* < 0.05 and VIP > 1), and 121 significantly changed compounds were identified in a volcano plot (Fig. 10B). Among these, 30 compounds in the FSD group were significantly upregulated compared to the SD group such as pyrocatechol, nicotinic acid and (S)-(-)-2-hydroxyisocaproic acid, and 91 compounds were significantly downregulated, including maleic acid, nicotinamide, and poricoic acid. The details are shown in Additional file 1.

**Fig. 11:**
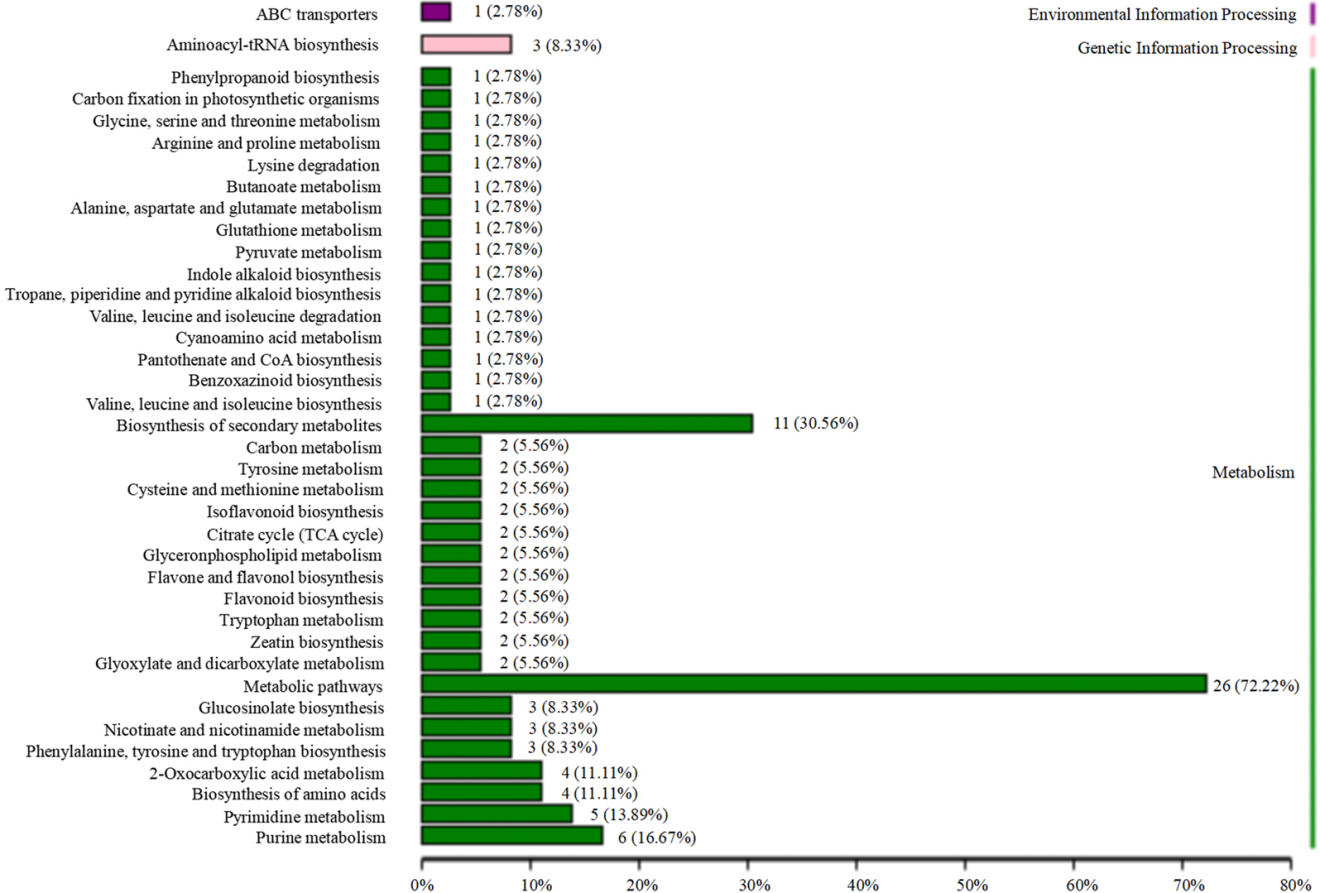
Kyoto Encyclopedia of Genes and Genomes (KEGG) pathway classification maps of significantly different compounds between Sijunzi decoction (SD) and fermented Sijunzi decoction (FSD). The number behind the pathway represents the amount of significantly different compounds in this pathway; the percentage indicates the ratio of amount of significantly different compounds in the pathway to all significantly different compounds

The results of functional annotation using Kyoto Encyclopedia of Genes and Genomes (KEGG) are shown in Fig. 11. Six pathways were annotated and determined to be related to more than four significantly different compounds, as follows: biosynthesis of secondary metabolites (11), metabolic pathways (26), 2-oxocarboxylic acid metabolism (4), biosynthesis of amino acids (4), pyrimidine metabolism (5), and purine metabolism (6). The details are shown in Additional file 2.

## Discussion

The gut microbiota is a complex ecosystem that carries out several essential functions, including carbohydrate metabolism, interaction with the immune system, and prevention against pathogen invasion [24]. Intestinal flora is susceptible to dysbacteriosis caused by external and internal factors, and antibiotics are one of the major causes [25]. As such, to observe the alleviation of TCM, LP, and FTCM on dysbacteriosis, we established a mouse dysbacteriosis model by intragastric administration of CS. Ceftriaxone is a broad-spectrum, third-generation cephalosporin that is widely used to treat gastrointestinal infections. Repeated overuse of this antibiotic can disrupt the equilibrium of the intestinal flora and cause side effects like AAD [26]. In the present study, increases in stool weight, total number of fecal output, and fecal water content induced by CS indicated a successful establishment of the diarrhea model in mice. Moreover, bacterial culture tests revealed the destruction of specific intestinal flora in mice gavaged with antibiotics, which was similar to the findings of the previous study [27]. Taken together, the above results are consistent with our previous study that found ceftriaxone and ciprofloxacin can cause watery diarrhea and intestinal microbial disorders in animal models [28].

An increasing body of evidence has shown that probiotics inhibit the proliferation of harmful bacteria in the intestine, promote the proliferation of beneficial bacteria, and effectively restore and balance the intestinal flora, which helps in the prevention and treatment of AAD [29]. Notably, previous studies reported that the spleen deficiency type occupies a major proportion of AAD, in response various tonifying Qi-invigorating TCMs are used to cure bowel diseases, treat intestinal flora disorder, enhance immunity, relieve fatigue, and prolong lifespan either as clinical medications or daily diets [7]. Qiweibaizhu powder has been used to effectively treat dysbiosis diarrhea by improving intestinal flora and promoting the reproduction of beneficial bacteria like *Bifidobacteria* and *Lactobacillius* [27, 30]. Therefore, probiotics combined with TCM has attracted more attention to balance the gut microbiota to reduce dysbiosis [31].

In this study, the water content of the above animal feces was significantly reduced after continuous treatment with TCM, LP, and FTCM for 7 days, in particular, FSD significantly constantly relieved diarrhea symptoms from 4th day to 7th day, and on 7th day, compared to SD, the fecal water content of mice was markedly decreased in the FSD group. The number of *Lactobacillus* and *Bifidobacterium* in mice treated with LP, FSD, and FSHD was significantly increased compared to the CS group, and the number of *Colibacillus* was significantly decreased, therefore, LP, FSD, and FSHD supported the growth of beneficial flora and cleared intestinal pathogenic bacteria. These findings may be attributable to the altered pH in FSD and FSHD which is in agreement with results of an earlier report in which fermentation-induced low gut pH inhibited the proliferation of *Colibacillus* [32]. Although BD and SD increased the number of beneficial bacteria, both treatments also promoted the growth of harmful bacteria (*Colibacillus*), showing that fermentation had more favourable effects than non-fermentation.

The intestine is one of the most important visceral organs, not only for digestion and absorption of nutrients, but also for its innate barrier that protects the body from pathogenic microorganisms. Small intestinal tissue can be assessed by HE staining. The observed severe damage to the small intestine chorionic villi and histomorphological changes suggest that small intestine absorption and barrier function are severely damaged [33]. Studies have shown that TCM has obvious advantages in the treatment of intestinal mucosal barrier dysfunction [34]. He et al. treated broilers with probiotics (*Bacillus subtilis*, *Bacillus licheniformis*, and *Saccharomyces cerevisiae*), which increased the VH/CD in the duodenum [35]. In addition, piglets treated with *LP 299v* had a lower incidence of diarrhea than the control group, the VH/CD in their jejunum and ileum increased, and the structure of their gut microbiota was altered [36]. After developing diarrhea, the water content in mouse intestines increased, which promoted the softening of feces and stimulated the intestinal mucosa, thus causing mucosal damage. In the present study, the villus height of the duodenum increased after mice were fed with LP, BD, SD, FBD, and FSD; additionally, the VH/CD of the duodenum was markedly increased after the administration of SD and FSD via gavage, similar to the findings of the above studies. In this study, the VH/CD of the jejunum was enhanced in the CS + SD, CS + FBD, and CS + FSD groups, and SHD treatment improved both villus height and the VH/CD of the ileum. Overall, the above treatments promoted intestinal growth and reduced the impact of diarrhea on the intestinal mucosa and epithelial microvilli.

Furthermore, intestinal epithelial cells are connected through tight junctions (TJ), which regulate the intestinal barrier permeability and epithelial integrity. Therefore, TJ proteins are essential for the maintenance of human health [37]. Moreover, ZO-1 and occludins are the most critical components in the structural and functional organization of TJ [38]. Accordingly, the gut flora targets various intracellular pathways, alters the expression and distribution of TJ proteins, and regulates intestinal barrier function [39]. AQPs are water-channel membrane proteins that are expressed in various tissues. Reportedly, at least seven AQP subtypes (AQPs 1, 3, 4, 5, 7, 8, 9, and 11) are expressed in the gastrointestinal tract and play important roles in various physiological and pathological processes [40]. In particular, the distal small intestine and proximal colon are the major sites of expression of AQPs 1, 3, and 4 [41]. Zhang et al. [20] observed that rats with AAD exhibited defective gastrointestinal integrity and improper epithelial organization, with decreased expression of aquaporin-encoding genes, aberrant TJ proteins, as well as the reduced number of goblet cells compared to control animals. Likewise, we found that the *AQP1* and *ZO-1* mRNA expression levels in the duodenum and colon were significantly attenuated in mice with diarrhea. In addition, these two gene expressions were enhanced in mice after treatment with LP, BD, SD, or FSD. These results coincided with our morphological observations in intestine.

Because FSD was superior to other treatments in most indicators, compounds in SD and FSD were detected by UHPLC-Q-TOF/MS. The results revealed that 30 compounds in FSD were significantly upregulated compared to SD, of which, the four were noteworthy, including (S)-(-)-2-hydroxyisocaproic acid, L-methionine, 4-guanidinobutyric acid (4GBA), and phenyllactate (PLA). (S)-(-)-2-hydroxyisocaproic acid, also known as L-leucine, is a signaling amino acid (AA) in animal metabolism that can elevate villus height in the duodenum and the VH/CD of the duodenum and ileum [42]. Therefore, the upregulated (S)-(-)-2-hydroxyisocaproic acid in FSD might partly attribute to the alleviation of the villus height and VH/CD in the duodenum of diarrhea mice in this study. In addition, L-methionine is an essential AA in humans and other vertebrates. It cannot be synthesized by the body and must be obtained from the diet. Methionine absorption from the gastrointestinal tract is highly efficient [43]. Studies have shown that dietary supplementation of methionine is beneficial to intestinal development and antioxidant function in pigs [44]. Altogether, (S)-(-)-2-hydroxyisocaproic acid and L-methionine may play a significant role in improving the intestinal health of mice with diarrhea. 4GBA is an alkaloid included in guanidino compounds, which inhibits the growth of *Helicobacter. pylori* in a dose-dependent manner and may be useful in the treatment and/or protection of gastritis [45]. Possessing the same functional group, 4-methylguanidine butyric acid inhibits harmful bacteria and fungi, such as *Staphylococcus aureus*, *Escherichia coli*, *Saccharomyces cerevisiae*, and *Aspergillus niger* [46]. In the present study, the high 4GBA in FSD might also play a part role in inhibiting the growth of *Colibacillus*. Importantly, PLA is found in various foods, such as honey, pickles, sourdough, and a variety of fermented foods [47]. It has versatile antimicrobial activity against food-borne pathogenic bacteria [48] and spoilage mold [49]. Furthermore, PLA has great potential for application in food, feeds, and pharmaceuticals. Therefore, increased PLA in FSD could optimize the compositions of intestinal flora in mice with AAD. These four compounds can potentially be utilized to inhibit harmful bacteria and to improve intestinal development.

## Conclusions

TCM, LP, and TCM fermented with LP alleviated diarrhea symptoms, regulated the gut flora, and maintained the integrity of the intestinal villi in mice exposed to 4 g/kg CS. Additionally, intestinal barrier function was also improved by TCM, LP, and FTCM through increased expressions of AQPs and TJs. Through UHPLC-Q-TOF/MS, four compounds including (S)-(-)-2-hydroxyisocaproic acid, L-methionine, 4GBA, and PLA in FSD were identified, which might play certain roles in modulating intestinal flora and improving villi structure. Collectively, these findings provide a theoretical basis for the further development and application of TCM, probiotics, and FTCM in clinical use with antibiotics.

## Supplementary Information

**Additional file 1.** The up-regulated and down-regulated differentially expressed compounds.

**Additional file 2.** The KEGG classification.

## Data Availability

The datasets used and/or analysed during the current study are available from the corresponding author on reasonable request.

## Abbreviations

BD: Buzhongyiqi decoction
SD: Sijunzi decoction
SHD: Shenlingbaizhu decoction
TCM: Traditional Chinese medicine
FTCM: Fermented traditional Chinese medicine
LP: *Lactobacillus plantarum*
AAD: Antibiotic-associated diarrhea
FSD: Fermented SD
CS: Ceftriaxone sodium
UHPLC-Q-TOF/MS: Ultra-high-performance liquid chromatography coupled with quadrupole time-of-flight mass spectrometry
AQPs: Aquaporins
TJ: Tight junction
PCA: Principal component analysis
OPLS-DA: Orthogonal partial least squares-discriminant analysis
KEGG: Kyoto Encyclopedia of Genes and Genomes
4GBA: 4-guanidinobutyric acid
PLA: Phenyllactate
